# SToRytelling to Improve Disease outcomes in Gout (STRIDE-GO): a multicenter, randomized controlled trial in African American veterans with gout

**DOI:** 10.1186/s12916-021-02135-w

**Published:** 2021-11-09

**Authors:** Jasvinder A. Singh, Amy Joseph, Joshua Baker, Joshua S. Richman, Terrence Shaneyfelt, Kenneth G. Saag, Seth Eisen

**Affiliations:** 1 Medicine Service, VA Medical Center, 700 19th St S, Birmingham, AL 35233 USA; 2 Department of Medicine at School of Medicine, Faculty Office Tower 805B, 510 20th Street S, Birmingham, AL 35294 USA; 3 Division of Epidemiology at School of Public Health, University of Alabama at Birmingham, 1720 Second Ave South, Birmingham, AL 35294-0022 USA; 4 Washington University School of Medicine, St. Louis, MO USA; 5 St. Louis Veterans Affairs Medical Center, St. Louis, MO USA; 6 Corporal Michael J. Crescenz Veterans Affairs Medical Center, Philadelphia, PA USA; 7 University of Pennsylvania, Philadelphia, PA USA; 8 Department of Surgery, University of Alabama at Birmingham, Birmingham, AL USA

**Keywords:** Gout, Adherence, Trial, Storytelling, Randomized controlled trial, Urate-lowering therapy, African American, Disparities, Race/ethnicity, Medication adherence

## Abstract

**Background:**

Urate-lowering therapy (ULT) adherence is low in gout, and few, if any, effective, low-cost, interventions are available. Our objective was to assess if a culturally appropriate gout-storytelling intervention is superior to an attention control for improving gout outcomes in African-Americans (AAs).

**Methods:**

In a 1-year, multicenter, randomized controlled trial, AA veterans with gout were randomized to gout-storytelling intervention vs. a stress reduction video (attention control group; 1:1 ratio). The primary outcome was ULT adherence measured with MEMSCap™, an electronic monitoring system that objectively measured ULT medication adherence.

**Results:**

The 306 male AA veterans with gout who met the eligibility criteria were randomized to the gout-storytelling intervention (*n* = 152) or stress reduction video (*n* = 154); 261/306 (85%) completed the 1-year study. The mean age was 64 years, body mass index was 33 kg/m^2^, and gout disease duration was 3 years. ULT adherence was similar in the intervention vs. control groups: 3 months, 73% versus 70%; 6 months, 69% versus 69%; 9 months, 66% versus 67%; and 12 months, 61% versus 64% (*p* > 0.05 each). Secondary outcomes (gout flares, serum urate and gout-specific health-related quality of life [HRQOL]) in the intervention versus control groups were similar at all time points except intervention group outcomes were better for the following: (1) number of gout flares at 9 months were fewer, 0.7 versus 1.3 in the previous month (*p* = 0.03); (2) lower/better scores on two gout specific HRQOL subscales: gout medication side effects at 3 months, 32.8 vs. 39.6 (*p* = 0.02); and unmet gout treatment need at 3 months, 30.9 vs. 38.2 (*p* = 0.003), and 6 months, 29.5 vs. 34.5 (*p* = 0.03), respectively.

**Conclusions:**

A culturally appropriate gout-storytelling intervention was not superior to attention control for improving gout outcomes in AAs with gout.

**Trial registration:**

Registered at ClinicalTrials.gov NCT02741700

**Supplementary Information:**

The online version contains supplementary material available at 10.1186/s12916-021-02135-w.

## Background

Gout, the most common inflammatory arthritis in adults [1], affects people with cardiovascular comorbidities and the elderly [1], making it a disease of at-risk populations. Gout leads to disproportionately higher disease burden in African Americans (AAs) compared to Caucasians: (1) gout prevalence and incidence are 1.3- and 1.7-fold higher [1, 2]; (2) allopurinol treatment rates were lower (odds ratio 0.18) [3]; (3) non-adherence to urate-lowering therapy (ULT; a key recommendation from all gout treatment guidelines) [4] was 2-fold higher [5]; and (4) baseline serum urate was higher (SU; 7.9 vs. 7.1), achievement of target SU < 6 mg/dl (odds ratio, 0.67) is lower [6]; and (5) rates of emergency room visits and hospitalizations for gout were 2.6 times higher [6]. Therefore, developing and testing interventions to improve ULT adherence in AAs with gout is a high-priority for research.

High rates of ULT non-adherence in gout are well-described [7, 8]. Medication adherence (Medication Possession Ratio (MPR) ≥ 80%) was lowest for gout among seven chronic conditions [9]. ULT adherence barriers include forgetfulness, perceived ULT side effects and patient knowledge gaps [10, 11]. Patients with greater understanding of their gout have higher ULT adherence [12]. Health belief barriers to ULT adherence may be addressed by behavioral interventions. Therefore, we developed a novel, culturally appropriate gout-storytelling intervention targeting ULT adherence for AAs, based on qualitative work with the target population [13].

The objective of this multicenter, randomized controlled trial (RCT) was to assess the efficacy of gout-storytelling intervention in improving ULT adherence (primary) and other gout outcomes (gout flares, serum urate, gout-specific health-related quality of life (HRQOL) and treatment satisfaction) in AA veterans with gout. We hypothesized that gout-storytelling would be superior to the control intervention in improving ULT adherence and other gout outcomes.

## Methods

### Study population, study sites, randomization, and ClinicalTrials.gov registration

We conducted a multicenter, parallel, 2-arm, open-label RCT comparing a culturally appropriate gout-storytelling intervention to a control intervention (stress reduction) in AA veterans with gout, recruited from the Birmingham, Philadelphia, and St. Louis VA Medical Centers. A stress reduction video of similar duration to the gout-storytelling intervention, narrated by the same AA veteran with gout, was chosen as our attention control. This ensured that control group members spent the same amount of time as the intervention group watching a power-point by the same narrator of the same race/ethnicity. Another reason for choosing stress reduction as that attention control is not known to be related to improved gout outcomes or ULT adherence. After obtaining informed consent, and after participants completed a 1-month run-in period, we randomized participants using a computer-generated randomization process based upon a permuted variable block design, stratified by study site and ULT MPR (< 80% or not). The study was registered at ClinicalTrials.gov (NCT02741700) and was approved by each of the participating sites’ Human Subjects Studies Programs. Veterans were provided $25 remuneration for completing each study assessment. The trial protocol provides additional details [14]. Patients or the public were not involved in the design, or conduct, or reporting, or dissemination plans of our research.

### Subject eligibility, recruitment, and retention

Adult AA veterans with gout diagnostic code (M10, M1A; 274.x, 274.xx) meeting the 1977 Preliminary American College of Rheumatology (ACR) classification criteria for gout [15], currently prescribed oral ULT (allopurinol or febuxostat; both once daily) for at least 6 months with low ULT adherence in the VA pharmacy records, defined as an average ULT (MPR < 80%) (# of outpatient oral ULT doses dispensed in the prior 180 days*100/180), were eligible. Participants who redistributed daily pills into a pillbox or were not currently prescribed oral ULT were excluded. Eligible subjects were mailed letters and contacted by phone by the research assistant at each site. Research assistants pre-screened eligible veterans for inclusion or exclusion using the pre-screening questionnaire (gout diagnosis, ULT prescribed, ACR gout classification criteria, AA race).

Those who passed the pre-screen were invited for a study enrollment visit, 2016–2019. The site PI confirmed study eligibility. After providing informed consent, participants used a touchpad to complete the baseline patient assessments on VA Research Electronic Data Capture (REDCap; Nashville, TN): (1) demographics: age, gender, income, marital status (covariates); (2) gout duration, baseline frequency of gout flares and baseline patient satisfaction with ULT treatment; (3) baseline gout-specific health-related quality of life (HRQOL) assessment using the gout assessment questionnaire gout impact scale (GAQ-GIS); (4) alcohol use (from the behavioral risk surveillance study’s [BRFSS] alcohol measure) [16] and body mass index (BMI); and (5) ULT non-adherence on Voils et al. self-reported questionnaire [17]. Baseline serum urate (SU) was drawn. Data were collected using the VA REDCap. To watch study DVDs at home at 2 and 4 months, free DVD players were provided to participants at the baseline visit. All participants received their 3-month ULT prescription in bottles with MEMSCap™ (an electronic cap monitoring system), which was dispensed at the baseline visit. Participants were instructed at the initial visit regarding the use of MEMSCap™ medication bottles and asked to bring them to the 3-, 6-, 9-, and 12-month study visits. Data were downloaded from the MEMSCap™ at each in-person study visit. If a participant missed a study visit, the coordinator arranged for a 90-day supply to be mailed and instructed the participant to transfer the new medication supply to the MEMSCap™ bottle. MEMSCap™ data were downloaded at the next in-person study visit. Reminder phone calls were made 2 days prior to each follow-up visit.

Two protocol modifications were made prior to study initiation: (1) based on the variable relationship between pharmacy record-based ULT MPR at baseline (3 vs. 6 vs. 12 months) and patient self-reported adherence during screening, we decided to add a 1-month run-in period prior to randomization using MEMSCap™ data and use MEMSCap™ ULT MPR as the measure of baseline adherence rather than pharmacy records; (2) we found that many patients had allopurinol MEMSCap™ ULT MPR of ≥ 80% during the 1-month run-in, suggesting the possibility of a Hawthorne effect on ULT adherence that would result in potentially excluding at-risk patients if < 80% adherence threshold was used an entry criterion. Therefore, we changed the inclusion criteria to enroll people regardless of their 1-month run-in period ULT MPR value, but also pre-specified analysis of all study outcomes stratified by baseline ULT MPR of < 80% vs. higher.

### Study intervention and control: development, pilot testing, and finalization of the gout-storytelling intervention and the stress reduction control intervention for AA veterans with gout

The intervention group received the gout storytelling video at baseline (in-person) in entirety during the clinic visit and was given a DVD to watch at home at study months 2 and 4. Storytelling in AA veterans’ own voices focused on stories about gout and its treatment, improving ULT adherence. In addition, the gout storytelling video included a pre-tested power-point slide presentation on gout (effects of disease, ULT, benefits of ULT adherence) narrated by an AA veteran with gout under the “Learn More” section of the DVD (disease manifestations, treatments) [13]. The intervention group also received a printed copy of the power-point presentation in the “Learn More” section at baseline. Each DVD intervention installment had additional new stories/clips (based on patient preference for the predominant message: diet [A], effect of disease [B], medication management [C]) and “Learn More” gout content narrated presentation. The intervention contained several gout “stories” (10 min) from multiple people with gout, and was vetted by the target population, AA veterans with gout.

The comparison condition was an attention control group identical to the intervention condition, aside from not including the gout-storytelling modules, and given a DVD to watch at home at study months 2 and 4. The video focused on stress management, adapted from the Centers for Disease Control (CDC) and narrated by the same veteran who narrated the gout PowerPoint presentation and was of the same length as the intervention videos (20 min each)*.* Participants in both groups were introduced to the MEMSCap™ and trained during their initial visit by research staff. DVDs and all intervention materials are available from the corresponding author.

### Primary study outcome

#### ULT adherence

We calculated ULT adherence over 3, 6, 9, and 12 months using the MEMSCap™ (Aprex Corp., Fremont, California), using the “percent doses taken correctly” measure that counted bottle cap opening once every 24 h as a success for ULT adherence (once daily medication). MEMSCap™ has a higher validity compared to other adherence measures (self-report, claims, etc.) [18], with excellent internal reliability [19], and high predictive validity [19].

### Secondary outcomes

Gout flares, gout-specific HRQOL, and self-reported ULT Adherence were assessed at 3-, 6-, 9-, and 12-month study visits. To reduce patient burden, we assessed patient satisfaction with treatment and serum urate at 6 and 12 months only and patient understandability of the intervention at 2 and 4 months only.

Patient-reported gout flares were assessed along with the total number of gout flares in the preceeding 1 and 2 months.

Self-reported ULT Adherence was assessed using a validated questionnaire by Voils et al. [17]. It has two scales that assess (1) the extent of non-adherence and (2) the reasons for non-adherence. Intraclass correlations were 0.58 for the extent score and ranged 0.07 to 0.64 for the reasons score.

Gout-specific health-related quality of life (HRQOL) was assessed with the Gout Impact scale (GIS) of the Gout assessment questionnaire (GAQ), a validated measure of specific impact of gout on HRQOL [20]. Clinically important difference on the GIS scale is between 5 and 8 points [21].

Patient satisfaction with treatment was assessed by Satisfaction with Medications Questionnaire (SATMED-Q) that has 17 items with total score ranging from 0 to 68, transformed to a 0 to 100 scale [22]. SATMED has six dimensions: treatment effectiveness, convenience of use, impact on daily activities, medical care, global satisfaction, and side effects.

SU was determined by an enzymatic uricase method manufactured by Stanbio Laboratory (Boerne, TX), a standardized assay [23]. SU was assessed at the baseline visit (i.e., at the end of run-in-period) and at the 6- and 12-month follow-up.

Patient Education Materials Assessment Tool for Audiovisual Materials (PEMAT-A/V) was used to assess the understandability, actionability, and potential impact of messages on change in behavior, including ULT adherence [24].

### Statistical analyses

Descriptive statistics for demographics (age, race, income, marital status, time since diagnosis of gout) and clinical parameters (ULT adherence, # gout flares, satisfaction, serum urate, GAQ) were obtained. All analyses followed intent-to-treat principles. For the primary outcome, ULT adherence, we conducted an unadjusted analysis using the two-sample *t* test. Ordinary least squares regression was used to test for treatment difference at 6 months adjusting for age, BMI, income, baseline MPR, baseline SU, gout duration, baseline gout flares, site, and alcohol use. We also evaluated for differences in treatment effect stratified by these characteristics.

Quasi-Poisson regression was used to test for group differences in the number of gout flares in the past month or past 2 months (assessed at 3, 6, 9, and 12 months) adjusting for covariates and accounting for over-dispersion. Finally, separate logistic regression models were used to measure treatment differences in the odds of achieving target serum urate < 6 mg/dl. To analyze the longitudinal data, we used generalized estimating equations (GEE) to model ULT adherence (continuous outcome at 3, 6, 9, and 12 months). As an exploratory analysis, regression trees were used to identify subgroups with differential efficacy of the intervention.

### Sample size and power

Assuming a standard deviation (SD) of 15%, very close to the SD of 14% reported by Briesacher et al. [9], 125 patients/group (total of 306 to account for 18% drop out rate) was expected to provide 80% power to detect an absolute difference between means of ULT adherence of 6% (equates with Cohen’s effect size of 0.40), assuming a control vs. intervention group ULT adherence of 55% vs. 61% [9], and using a two-tailed type I error rate of 0.05.

## Results

### Study participant characteristics

**Fig. 1:**
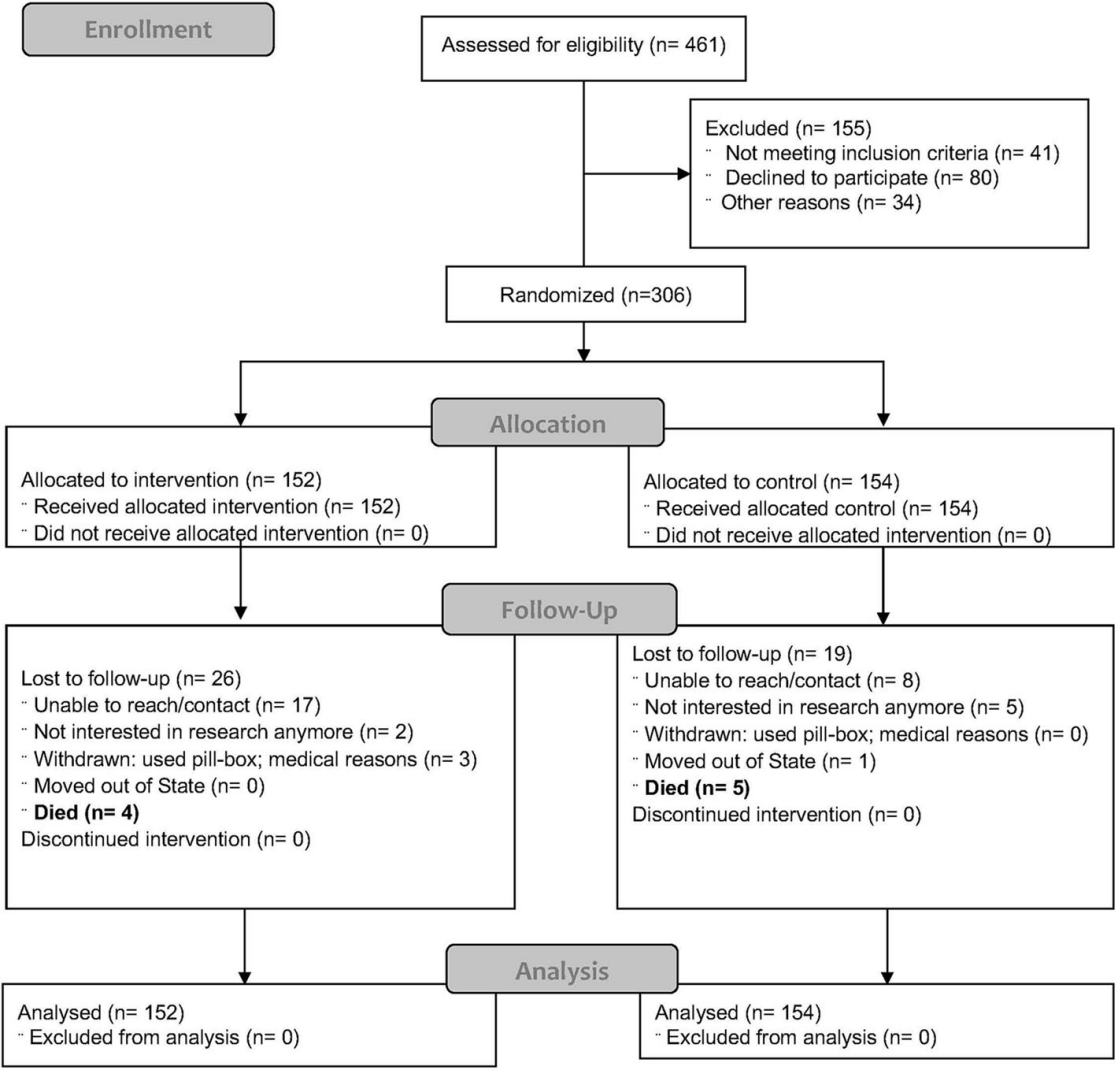
Study participant CONSORT diagram

Study participant characteristics are provided in Table 1. Of the 461 AA male veterans with gout screened, 306 met the eligibility criteria, randomized to the gout-storytelling intervention (*n* = 152) or the control intervention (*n* = 154; stress reduction). Participant characteristics were evenly distributed across the study arms. The mean age was 64 years, mean BMI was 33, mean gout disease duration was 3 years, and > 30% had incomes ≥ $60,000 (Table 1). The mean baseline MPR for ULT was 89% versus 87% respectively in the run-in study phase. At baseline, gout flares in the treatment vs. control groups were similar 1.4 versus 1.4 in the last month, and 2.3 versus 2.5 in the last two months, respectively (Table 1). The study consort diagram is shown in Fig. 1; 261/306 (85%) completed the study and similar numbers of people were lost to follow-up in each study arm.

**Table 1:** Demographic and clinical features of the patients at baseline

	Gout storytelling ***N*** = 152	Control ***N*** = 154	***p*** value
	***N*** (%) or mean (SD)	***N*** (%) or mean (SD)	
Age, in years	64.0 (8.3)	65.0 (8.0)	0.29
AA race/ethnicity	152 (100%)	154 (100%)	1.0
Marital status			
Single	38 (26%)	37 (25%)	0.39
Married	59 (40%)	65 (43%)	
Divorced	30 (20%)	32 (21%)	
Widowed	9 (6%)	10 (7%)	
Separated	13 (9%)	6 (4%)	
Missing	3 (2%)	4 (3%)	
Annual income, US $			
< $20,000	0 (0%)	0 (0%)	0.90
$20,000–$39,999	53 (37%)	57 (38%)	
$40,000–$59,999	44 (31%)	45 (30%)	
$60,000–$99,999	28 (20%)	26 (17%)	
$100,000–$149,999	13 (9%)	16 (11%)	
$150,000 and above	4 (3%)	5 (3%)	
Missing	10 (7%)	5 (3%)	
Body mass index (BMI), kg/m^2^	32.9 (5.7)	32.9 (8.0)	0.98
Gout duration, in years	3.1 (1.0)	3.0 (0.9)	0.43
ACR/EULAR 2015 Gout classification criteria score^a^	9.7 (4)	9.0 (3.4)	0.07
**Primary outcome**			
Mean ULT MPR with MEMSCap™	89% (14%)	87% (16%)	0.36
% With MPR ≥ 80% with MEMSCap™	124 (84%)	119 (78%)	0.29
**Secondary outcomes**			
Voils self-reported non-adherence scale^b^	1.60 (0.8)	1.40 (0.7)	**0.03**
Proportion with pt.-reported current gout flare	27 (18%)	30 (20%)	0.79
Serum urate, mg/dl	5.90 (1.8)	5.70 (1.7)	0.58
SATMED side effects	14 (9.4%)	13 (8.8%)	1.0
SATMED subscales			
Treatment effectiveness (0–100)	8.15 (3.2)	8.12 (3.7)	0.94
Convenience of use (0–100)	9.37 (3.3)	9.54 (3.2)	0.65
Impact on daily living/activities (0–100)	8.85 (2.4)	8.55 (3.7)	0.43
Medical care (0–100)	5.97 (2.4)	5.81 (2.5)	0.57
Undesirable side effects (0–100)	11.69 (1.1)	11.53 (1.7)	0.35
Global satisfaction (0–100)	10.36 (2.1)	9.69 (2.9)	**0.02**
Total SATMED composite score (0–100)	79.98 (14.6)	78.29 (19.4)	0.39
Gout-specific HRQOL on GAQ-GIS subscales			
Gout concern overall	52.22 (27)	48.49 (27.2)	0.23
Gout medication side effects	39.14 (24.3)	39.38 (24.7)	0.94
Unmet gout treatment need	36.32 (18.8)	34.97 (20.3)	0.55
Well-being during attack	47.25 (25.5)	45.81 (25.3)	0.62
Gout concern during attack	55.58 (23.8)	49.58 (25.7)	**0.03**
PEMAT-A/V			
Understandability (items 1–13)	92.16 (12.5)	91.26 (15.7)	0.58
Accountability (items 14–17)	91.12 (16.6)	88.64 (21.0)	0.25
BRFSS alcohol use in last 30 days			
Days per week with ≥ 1 alcohol drinks	1.61 (3.5)	1.95 (4.2)	0.43
Days per month with ≥ 1 alcohol drinks	3.68 (9.9)	3.66 (6.4)	0.98
Alcohol drinks per day, median (interquartile range)^c^	0 (0, 2)	0 (0, 2)	0.56
Alcohol drinks ≥ 5 alcohol drinks	0.81 (2.8)	0.91 (3.2)	0.78
Largest number of alcohol drinks on any occasion	1.72 (3.7)	1.4 (2.1)	0.36
Baseline gout flares			
Last/previous 1 month	1.40 (1.9)	1.40 (2.8)	0.88
Last/previous 2 month	2.30 (3.7)	2.50 (5.5)	0.79
Pill count	28.0 (44.5)	30.7 (49.7)	0.79

^a^All patients met the 1977 Preliminary American College of Rheumatology (ACR) gout classification criteria; 87% in each group also met the 2015 ACR-European League Against Rheumatism (EULAR) criteria (a total score of 8 or higher or urate crystals present in the synovial/joint fluid or in tophus)

^b^Self-reported non-adherence scale by Voils et al.; the total score reflects the respondents’ agreement with three ordinal scaled items about non-adherence, ranging from 1 (strongly disagree) to 5 (strongly agree); ^c^median (interquartile range) provided for alcohol drinks/day, due to extremely high values for some individuals, which makes the median a better measure of central tendency than the mean

*AA* African American, *ULT* urate-lowering therapy, *MPR* Medication Possession Ratio, *SATMED* Satisfaction with Medications Questionnaire, *PEMAT-A/V* Patient Education Materials Assessment Tool for Audiovisual Materials, *SU* serum urate, *HRQOL* health-related quality of life, *GAQ-GIS* Gout Impact scale of the Gout assessment questionnaire, *BRFSS* Behavioral Risk Factor Surveillance System

Significant *p* values are in bold; All *p*-values are based on student's t-test except for the median alcohol drinks/day, which is based on the Wilcoxon signed-rank test

### Primary outcome: ULT MPR with MEMSCap™

**Fig. 2:**
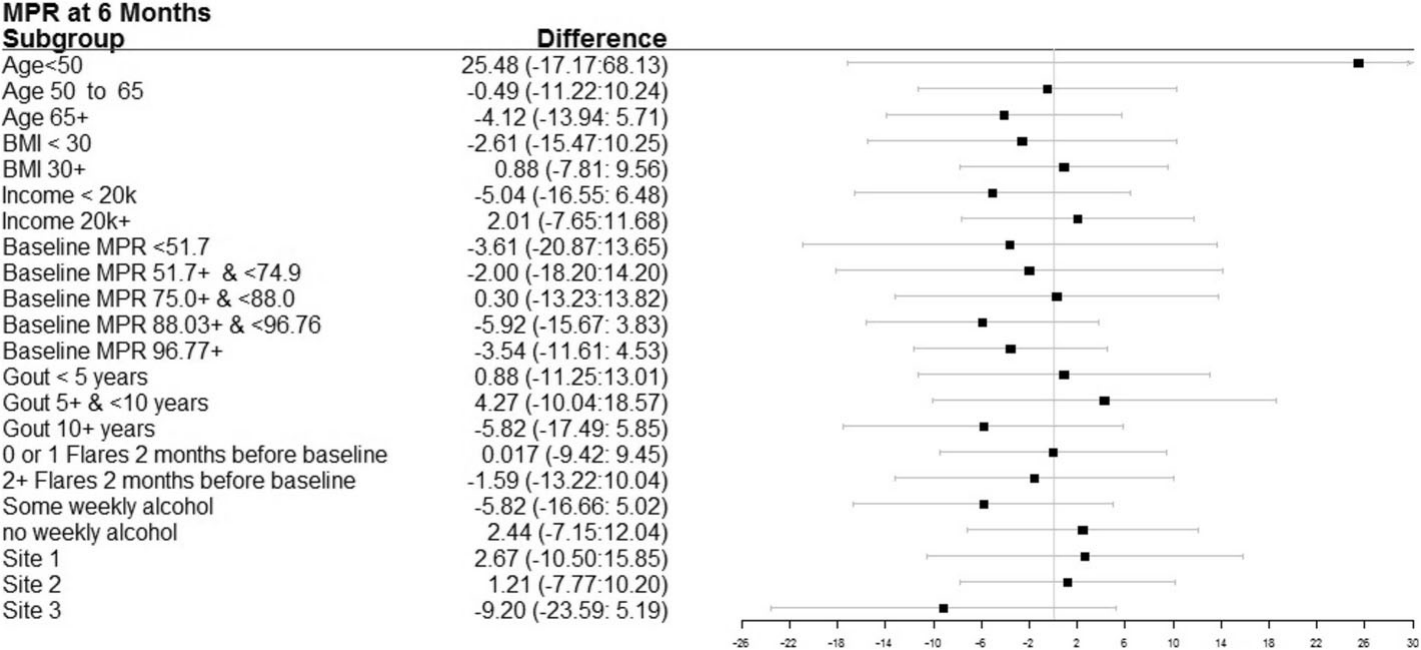
Treatment vs. control group differences in primary outcome of ULT MPR by pre-specified subgroups. Point estimates are presented as squares and the whiskers represent the 95% confidence intervals

The MPR was similar in the intervention vs. control groups at each time point (Table 2). Subgroup analyses did not reveal significant differences between treatment arms (Fig. 2). GEE models with longitudinal data analyses (3, 6, 9, and 12 months) showed no significant differences between groups (data available on request).

**Table 2:** Primary and key secondary study outcomes

	Gout storytelling ***N*** = 152	Control ***N*** = 154	Difference (95% CI)	***p*** value
	***N*** (%) or estimate (95% CI)	***N*** (%) or estimate (95% CI)		
**Primary outcome; MEMSCap™ MPR**				
Baseline	75.91 (71.71; 80.11)	71.90 (67.75; 76.06)	4.01 (−1.90; 9.91)	0.18
3 months	72.61 (67.88; 77.35)	70.12 (65.49; 74.75)	2.49 (−4.13; 9.12)	0.46
6 months	68.52 (63.40; 73.64)	69.33 (64.36; 74.30)	-0.81 (−7.95; 6.33)	0.82
9 months	65.85 (60.14; 71.55)	67.32 (61.84; 72.80)	−1.48 (−9.39; 6.44)	0.72
12 months	60.54 (54.29; 66.79)	63.82 (57.83; 69.82)	−3.29 (−11.95; 5.38)	0.46
**Key secondary outcomes**				
**1. Gout flares in last 1 month**				
Baseline	1.38 (0.98; 1.77)	1.42 (1.03; 1.81)	−0.044 (−0.60; 0.51)	0.88
3 months	1.29 (0.73; 1.85)	1.53 (0.98; 2.07)	−0.24 (−1.02; 0.54)	0.55
6 months	1.32 (0.82; 1.83)	1.55 (1.06; 2.03)	−0.23 (−0.93; 0.48)	0.53
9 months	**0.74 (0.40; 1.08)**	**1.27 (0.95; 1.59)**	−**0.53 (**−**0.99;** −**0.06)**	**0.03**
12 months	0.74 (0.45; 1.02)	0.99 (0.72; 1.26)	−0.26 (−0.65; 0.13)	0.20
**2. Voils self-reported ULT non-adherence**				
Baseline	1.57 (1.46; 1.69)	1.39 (1.28; 1.50)	0.18 (0.02; 0.34)	**0.03**
3 months	1.49 (1.38; 1.61)	1.46 (1.34; 1.57)	0.04 (−0.13; 0.20)	0.66
6 months	1.45 (1.33; 1.57)	1.45 (1.33; 1.57)	0.01 (−0.16; 0.17)	0.97
9 months	1.40 (1.28; 1.52)	1.48 (1.36; 1.59)	−0.08 (−0.24; 0.09)	0.36
12 months	1.46 (1.32; 1.59)	1.46 (1.33; 1.60)	−0.01 (−0.20; 0.18)	0.94
**3. Total SATMED composite score (0–100)**				
Baseline	79.98 (77.26; 82.71)	78.29 (75.59; 81.00)	1.69 (−2.16; 5.53)	0.39
6 months	63.74 (59.01; 68.47)	66.28 (61.58; 70.98)	−2.54 (−9.21; 4.12)	0.45
12 months	62.16 (57.09; 67.23)	67.43 (62.39; 72.47)	−5.27 (−12.42; 1.88)	0.15
**4. Serum urate, mg/dl**				
Baseline	5.85 (5.56; 6.13)	5.73 (5.45; 6.01)	0.11 (−0.28; 0.51)	0.57
6 months	6.10 (5.64;6.56)	5.79 (5.38; 6.20)	0.31 (−0.30; 0.93)	0.47
12 months	5.94 (5.61; 6.27)	5.72 (5.41; 6.04)	0.22 (−0.23; 0.68)	0.34
**5. Serum urate,** ≤ **6 mg/dl**				
Baseline	88 (59.5%)	91 (60.3%)	0.97 (0.61; 1.54)	0.89
6 months	36 (52.9%)	54 (63.5%)	0.65 (0.34; 1.24)	0.19
12 months	67 (56.3%)	79 (60.8%)	0.83 (0.50; 1.38)	0.47
**6. Serum urate,** ≤ **5 mg/dl**				
Baseline	53 (35.8%)	58 (38.4%)	0.89 (0.56; 1.43)	0.64
6 months	20 (29.4%)	30 (35.3%)	0.76 (0.38; 1.52)	0.44
12 months	42 (35.3%)	49 (37.7%)	0.90 (0.54; 1.51)	0.69
**7. Gout-specific HRQOL on GAQ-GIS subscales**				
**3 months**				
Gout concern overall	47.40 (42.95; 51.86)	46.52 (42.11; 50.92)	0.89 (−5.38; 7.15)	0.78
Gout medication side effects	37.69 (33.29; 42.08)	40.69 (36.35; 45.04)	−3.01 (−9.19; 3.17)	0.34
Unmet gout treatment need	**30.85 (27.42; 34.27)**	**38.20 (34.81; 41.59)**	−**7.35 (**−**12.17;** −**2.54)**	**0.003**
Well-being during attack	**49.85 (45.37; 54.32)**	**40.06 (35.67; 44.45)**	**9.79 (3.52; 16.05)**	**0.002**
Gout concern during attack	51.60 (46.96; 56.25)	45.67 (41.07; 50.26)	5.94 (−0.60; 12.47)	0.08
**6 months**				
Gout concern overall	44.44 (39.84; 49.05)	44.21 (39.79; 48.62)	0.24 (−6.14; 6.61)	0.94
Gout medication side effects	**32.84 (28.62; 37.06)**	**39.60 (35.55; 43.65)**	−**6.76 (**−**12.61;** −**0.91)**	**0.02**
Unmet gout treatment need	**29.50 (26.29; 32.71)**	**34.52 (31.44; 37.60)**	−**5.02 (**−**9.47;** −**0.57)**	**0.03**
Well-being during attack	42.35 (37.61; 47.09)	41.47 (36.92; 46.02)	0.88 (−5.69; 7.45)	0.79
Gout concern during attack	47.27 (42.96; 51.59)	45.71 (41.57; 49.85)	1.56 (−4.42; 7.54)	0.61
**9 months**				
Gout concern overall	57.22 (52.83; 61.62)	58.32 (54.16; 62.48)	−1.09 (−7.14; 4.96)	0.72
Gout medication side effects	52.15 (47.85; 56.46)	51.10 (47.03; 55.18)	1.05 (−4.88; 6.98)	0.73
Unmet gout treatment need	43.24 (39.08; 47.40)	47.61 (43.67; 51.55)	−4.37 (−10.10; 1.36)	0.14
Well-being during attack	41.11 (36.05; 46.17)	39.97 (35.24; 44.70)	1.14 (−5.79; 8.07)	0.75
Gout concern during attack	51.59 (47.24; 55.94)	51.62 (47.50; 55.74)	−0.036 (−6.03; 5.95)	0.99
**12 months**				
Gout concern overall	40.97 (36.17; 45.76)	42.43 (37.84; 47.02)	−1.47 (−8.10; 5.17)	0.66
Gout medication side effects	33.20 (28.87; 37.52)	36.18 (32.04; 40.33)	−2.99 (−8.98; 3.00)	0.33
Unmet gout treatment need	28.55 (25.66; 31.44)	31.83 (29.06; 34.60)	−3.28 (−7.28; 0.73)	0.11
Well-being during attack	44.32 (39.24; 49.40)	38.34 (33.49; 43.18)	5.99 (−1.03; 13.00)	0.10
Gout concern during attack	45.59 (41.03; 50.16)	43.37 (39.01; 47.74)	2.22 (−4.10; 8.54)	0.49
**% with current gout flare**				
Baseline	27 (17.8%)	30 (19.6%)	0.89 (0.50; 1.58)	0.68
1 month	9 (6.5%)	20 (13.4%)	0.45 (0.20; 1.02)	0.06
3 months	19 (14.3%)	27 (19.4%)	0.69 (0.36; 1.31)	0.26
6 months	12 (9.5%)	20 (14.5%)	0.62 (0.29; 1.33)	0.22
9 months	14 (11.6%)	18 (13.2%)	0.86 (0.41; 1.81)	0.69
12 months	9 (7.4%)	13 (9.8%)	0.74 (0.30; 1.79)	0.50

*ULT* urate-lowering therapy, *MPR* Medication Possession Ratio, *SATMED* Satisfaction with Medications Questionnaire, *HRQOL* health-related quality of life, *GAQ-GIS* Gout Impact scale of the Gout assessment questionnaire

The proportion with MEMSCap™ MPR ≥ 80% was also similar in two groups: 3 months, 51.5% versus 50.0%; 6 months, 50.4% versus 47.4%; 9 months, 48.3% versus 47.7%; and 12 months, 42.4% versus 46.1% (*p* > 0.05 for each time).

### Pre-specified subgroup analyses including those by baseline MPR

For people with baseline ULT MPR < 80% during the run-in period, there were no significant differences between the treatment arms in primary or secondary outcomes (Additional file 1). There were no significant differences in primary or secondary outcomes in people with baseline ULT MPR ≥ 80%, except two GAQ-GIS subscale scores (Additional file 2).

### Secondary outcomes: gout flares, self-reported ULT adherence, serum urate, satisfaction with treatment, and gout-specific HRQOL

**Fig. 3:**
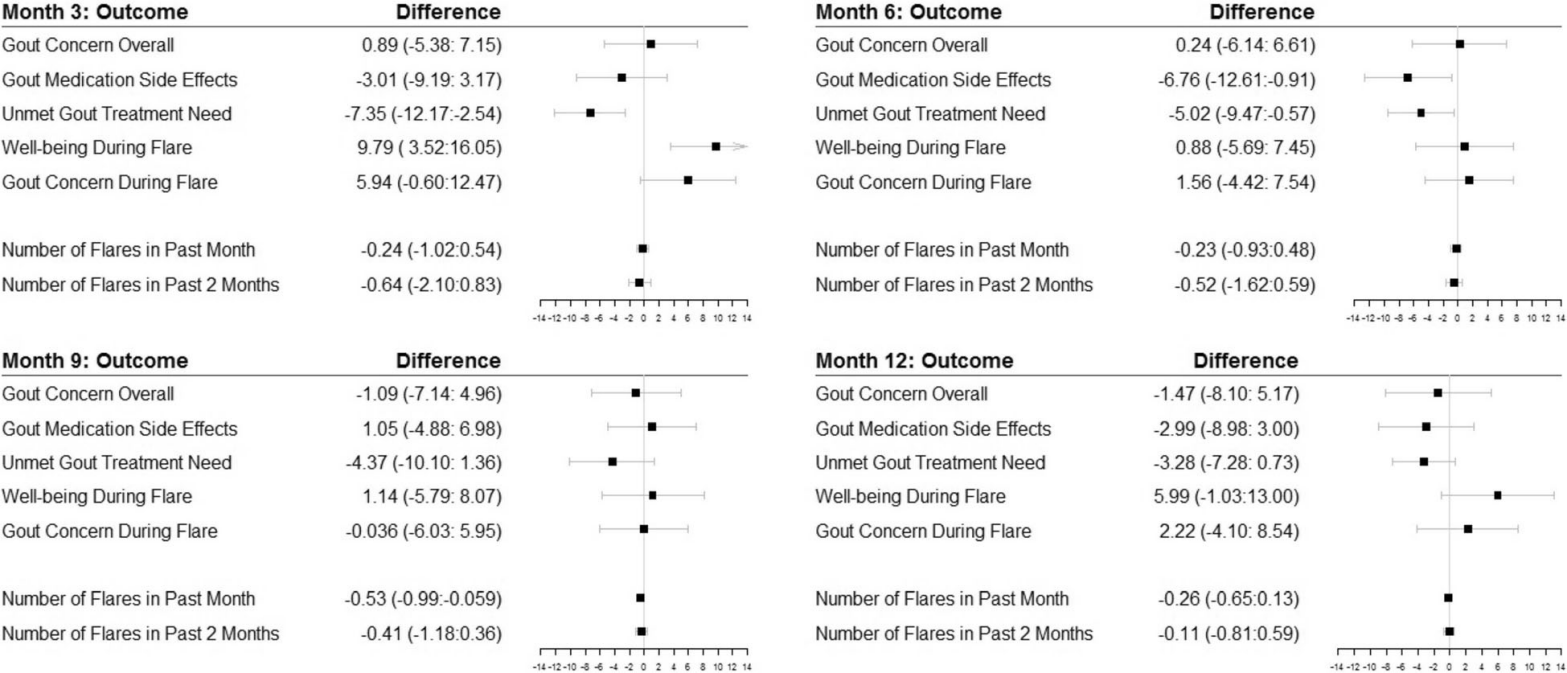
Treatment vs. control group differences in gout-specific HRQOL and gout flares in the last month at each study time point. Point estimates are presented as squares and the whiskers represent the 95% confidence intervals

The number of gout flares in the previous month declined over time and was similar in the treatment versus the control group except a significant difference in flares at 9 months, 0.7 versus 1.3 (*p* = 0.03) (Table 2; Fig. 3). This difference continued to be significant in the adjusted quasi-Poisson model. Patient-reported adherence to ULT on the Voils questionnaire, patient satisfaction with treatment, SU levels, and the proportion of people with SU at ≤ 6 mg/dl or ≤ 5 mg/dl were similar in the treatment versus the control group at all time-points (Table 2).

Gout-specific HRQOL scores on GAQ-GIS were also similar in the treatment versus  the control group except for statistically significant lower/better scores in gout medication side effects subscale scores at 3 months and unmet gout treatment need at 3 and 6 months, in the treatment group that also met the clinically meaningful difference threshold [21] (Table 2; Fig. 3). PEMAT-A/V total score and actionability subscale scores were significantly higher in the intervention vs. the control group (Table 3).

**Table 3:** Satisfaction with medication using the SATMED-Q subscales at 6 and 12 months and PEMAT-A/V scores at 2 months and 4 months post-intervention

	**Gout storytelling** ***N*** **= 152**	**Control** ***N*** **= 154**	**Difference (95% CI)**	***p*** **value**
	**Estimate (95% CI)**	**Estimate (95% CI)**		
SATMED-Q subscales; 6 months				
Total score (0–100)	63.74 (59.01; 68.47)	66.28 (61.58; 70.98)	−2.54 (−9.21; 4.12)	0.45
Undesirable side effects (0–100)	9.26 (8.55; 9.97)	9.56 (8.86; 10.27)	−0.30 (−1.30; 0.70)	0.55
Treatment effectiveness (0–100)	4.55 (4.03; 5.08)	4.58 (4.06; 5.10)	−0.025 (−0.77; 0.72)	0.95
Convenience of use (0–100)	7.59 (6.86; 8.31)	8.62 (7.89; 9.34)	−1.03 (−2.06; 0.00)	0.05
Impact on daily living/activities (0–100)	7.57 (6.88; 8.27)	7.83 (7.14; 8.52)	−0.26 (−1.24; 0.72)	0.60
Medical care (0–100)	5.28 (4.78; 5.78)	5.31 (4.81; 5.80)	−0.03 (−0.73; 0.68)	0.94
Global satisfaction (0–100)	9.09 (8.42; 9.77)	9.18 (8.50; 9.85)	−0.08 (−1.04; 0.87)	0.86
SATMED-Q subscales: 12 months				
Total score (0–100)	62.16 (57.09; 67.23)	67.43 (62.39; 72.47)	−5.27 (−12.42; 1.88)	0.15
Undesirable side effects (0–100)	8.95 (8.22; 9.68)	9.77 (9.05; 10.50)	−0.83 (−1.86; 0.21)	0.12
Treatment effectiveness (0–100)	4.41 (3.91; 4.91)	5.12 (4.62; 5.61)	−0.71 (−1.41; 0.00)	0.05
Convenience of use (0–100)	7.71 (6.99; 8.43)	8.39 (7.67; 9.11)	−0.68 (−1.70; 0.34)	0.19
Impact on daily living/activities (0–100)	7.62 (6.92; 8.33)	8.08 (7.38; 8.79)	−0.46 (−1.45; 0.54)	0.37
Medical care (0–100)	4.96 (4.45; 5.47)	5.25 (4.75; 5.76)	−0.29 (−1.01; 0.43)	0.43
Global satisfaction (0–100)	8.62 (7.92; 9.31)	9.23 (8.54; 9.92)	−0.62 (−1.60; 0.37)	0.22
	***N*** **(%)**	***N*** **(%)**	**Difference (95% CI)**	***p*** **value**
SATMED-Q side effect—yes				
6 months	9 (7.2%)	10 (7.4%)	0.97 (0.38; 2.47)	0.95
12 months	9 (7.6%)	5 (3.8%)	2.06 (0.67; 6.34)	0.21
PEMAT-A/V total	**Estimate (95% CI)**	**Estimate (95% CI)**	**Difference (95% CI)**	
Baseline	66.80 (64.85; 68.76)	65.61 (63.67; 67.55)	1.19 (−1.56; 3.95)	0.40
2 months	**76.23 (71.19; 81.28)**	**68.48 (63.47; 73.49)**	**7.75 (0.65; 14.86)**	**0.03**
4 months	64.64 (58.37; 70.91)	58.96 (52.72; 65.19)	5.68 (−3.16; 14.53)	0.21
PEMAT-A/V understandable subscale score				
Baseline	92.16 (89.90; 94.41)	91.26 (89.02; 93.50)	0.90 (−2.28; 4.07)	0.58
2 months	84.16 (78.61; 89.71)	76.22 (70.71; 81.74)	7.94 (0.11; 15.76)	0.05
4 months	71.66 (64.74; 78.58)	65.73 (58.86; 72.61)	5.93 (−3.83; 15.69)	0.24
PEMAT-A/V actionable subscale score				
Baseline	91.12 (88.10; 94.13)	88.64 (85.64; 91.63)	2.48 (−1.77; 6.73)	0.25
2 months	**83.72 (78.06; 89.37)**	**75.16 (69.54; 80.78)**	**8.55 (0.58; 16.53)**	**0.04**
4 months	69.57 (62.65; 76.49)	64.45 (57.57; 71.32)	5.12 (−4.63; 14.88)	0.30

The SATMED-Q contains 17 items, each scored on a 5-point Likert scale. The total composite score ranges between 0 and 68. The score was converted to a percentage as recommended by the author of the original version (=(raw score*100)/68); higher score = more satisfaction with medication

SATMED-Q, Satisfaction with Medications Questionnaire; PEMAT-A/V, Patient Education Materials Assessment Tool for Audiovisual Materials

Regression trees for MPR at 6 months and change in MPR from baseline to 6 months did not identify any specific subgroups in which the intervention was effective (Additional files 3 and 4: Fig. S1 and S2); similar observations were made for MPR at 12 months (data available on request).

## Discussion

Storytelling improved hypertension outcomes [25] and HPV vaccination [26]. Therefore, we examined gout storytelling-intervention, since gout is an excellent model for chronic intermittently symptomatic diseases. In this multicenter RCT in 306 AA male veterans with gout, a gout-storytelling behavioral intervention was not superior to a control intervention (stress reduction) in improving gout outcomes. ULT MPR by MEMSCap™ and most of the secondary outcomes were similar between the two groups. Slightly fewer gout flares, better gout-specific HRQOL scores (clinically and statistically) and a better actionability of the audiovisual materials were observed in the gout-storytelling vs. the control intervention; rates of missing observations were similar between groups. Several findings merit further discussion.

A key study strength was that the barriers and facilitators to optimal ULT adherence in our qualitative study with the target population (AA male veterans with gout) [13] and another study in minorities [27] mapped to the HBM model by Rosenstock [28]. The conceptual model for our gout storytelling intervention was based on narrative communication theory, developed by Slater [29] to deliver health belief messages constructed on the foundation of narrative communication appeals to the human affinity for “storytelling.” Its effectiveness in changing attitudes and behavior follows from the ability to reduce cognitive resistance through transportation (absorption in the story line) and identification with characters in the narrative. Other strengths of our storytelling-intervention are that it (1) was culturally appropriate for AA veterans; (2) was vetted by the target population, AA veterans with low ULT adherence, as being persuasive; (3) employed a randomized design; (4) adopted an “attention control video” to ensure that any effect observed was due to the gout storytelling intervention; (5) had an objective primary medication adherence outcome and clinically important and patient-relevant secondary outcomes (flares, treatment satisfaction, SU, HRQOL); and (6) included a 12-month follow-up period.

So, why did a culturally appropriate storytelling intervention fail, despite being based on a theoretical model [28] and a previous positive evidence in AAs with hypertension? [25]. Several factors may have contributed. First, the baseline ULT MPR in the run-in period was quite high with 51% of the participants with ULT MPR ≥ 80%. We hypothesized that this was a Hawthorne effect (and therefore modified the study inclusion criteria to allow participants with ULT MPR ≥ 80%). The observed reduction of ULT MPR from 72–76% at baseline vs. 60–64% at 12 months supported our hypothesis. The low mean baseline SU of 5.7–5.9 mg/dl further supports a high ULT adherence at enrollment. This indicates that future behavioral intervention studies in gout should consider requiring a minimum serum urate, for example, of more than > 6 or > 8 mg/dl to ensure enrolling people with inadequately controlled gout. The intervention may not have been intensive enough, since patients had a single observed intervention with potential, unconfirmed repeat DVD intervention dose at 2 and 4 months. DVDs were provided to the participants, but whether and to what extent they watched them could not be determined. In retrospect, the follow-up video should have been provided to participants as a weblink with login to watch at home or viewed by participants again at the 3-month face-to-face visit rather than as a DVD. This would have captured viewing frequency and duration for each patient, which would have provided adherence rates for repeat viewing at home and allowed analyses by “video-viewing dose” effect. Finally, despite developing a culturally appropriate, acceptable, and feasible gout-storytelling intervention, such a patient-focused intervention might simply be ineffective in this population.

We noted some evidence for efficacy of the storytelling intervention compared to the control, i.e., the effect on gout flares, gout-specific HRQOL, and actionability of the A/V content of the gout-storytelling intervention. Therefore, it may be worthwhile to examine this intervention further, perhaps among high-risk minority populations with gout, such as those with very low ULT adherence, frequent gout flares (≥ 2 per year) or frequent ER visits or hospitalizations due to gout flares.

Our study findings should be interpreted considering study limitations. Our findings are generalizable to AA veterans with gout, but not to women or non-veterans with gout. We did not assess the educational level of veterans in our study participants; in a previous publication by other investigators, education was reportedly similar between veterans and non-veterans [30]. Other characteristics such as income were assessed; 31% of our study participants had annual income of ≥ $60,000. Due to the concerns based on initial review of the ULT MPR pharmacy data, we modified the protocol to require a run-in period with MEMSCap™ and enrolled people with relatively high baseline ULT MPR. The potential for a Hawthorne effect due to the use of MEMSCap™ in the short run-in period may have contributed to challenges with the interpretation of the effect of the intervention vs. control.

## Conclusions

In conclusion, a culturally appropriate gout-storytelling intervention was not associated with an improvement in ULT adherence or other gout outcomes compared to a control intervention, in a multicenter RCT in AA male veterans with gout. The lack of effect of the effectiveness of a culturally appropriate gout-storytelling intervention may be due to a high baseline ULT MPR adherence and a potentially weak intervention, which likely required more than a single dose in an office setting—confirmation of repeat viewing using weblink and registration of patient viewing would have quantified adherence to the repeat dose of the intervention. Potential effect on gout flare rates, gout-specific HRQOL, and audio-visual material acceptability of an intervention that has already been developed and is available for use indicates that future studies in higher-risk, more symptomatic populations, or those with lower adherence to ULT are needed.

## Supplementary Information

**Additional file 1..** Primary and secondary RCT outcomes, for people with baseline MEMSCap™ ULT MPR <80% during the study run-in period. The additional file provides primary and secondary outcomes data for people with low baseline ULT MPR, comparing gout storytelling to stress reduction ‘control’ intervention.

**Additional file 2..** Primary and secondary RCT outcomes, for people with baseline MEMSCap™ ULT MPR ≥80% during the study run-in period. The additional file provides primary and secondary outcomes data for people with high baseline ULT MPR, comparing gout storytelling to stress reduction ‘control’ intervention.

**Additional file 3:.** Fig. S1. Regression tree for ULT MPR at 6 months. Regression tree results for ULT MPR at 6 months. The tree was allowed to consider all baseline variables that were used in any analyses. The goal was to identify whether there were any subgroups in which the intervention was efficacious. This would have been indicated by the appearance of ‘group’ at one of the split points in the tree. Our regression tree does not show ‘group’ at any split points in the tree. To interpret; each oval contains the mean MPR in that group (top number) and percent of the cohort (bottom number). The oval at the top of the graph indicates an overall mean MPR of 69% among the whole cohort (100%). Each split represents a dichotomization of the data with ‘yes’ on the left and ‘no’ on the right. So, the first split was chosen by the algorithm as baseline MPR < 78 (bl_mpr < 78), those meeting the condition (‘yes’, on the left) had a mean MPR of 51 and comprised 45% of the cohort; those with baseline MPR ≥78 had a mean MPR of 83 and represented the remaining 55% of the cohort. On the left side, those with baseline MPR <78 were then further subdivided by baseline MPR <55. Those meeting this condition represented 20% of the cohort with a mean MPR of 39 vs. the 24% of the cohort with baseline MPR ≥55 (but less than 78) with a mean 6-month MPR of 62.

**Additional file 4:.** Fig. S2. Regression tree for change in ULT MPR at 6 months. Regression tree results for change in ULT MPR from baseline to 6 months, with negative change indicating a decrease in MPR. The tree was allowed to consider all baseline variables that were used in any analyses. The goal was to identify whether there were any subgroups in which the intervention was efficacious. This would have been indicated by the appearance of ‘group’ at one of the split points in the tree. Our regression tree does not show ‘group’ at any split points in the tree. To interpret; each oval contains the mean MPR in that group (top number) and percent of the cohort (bottom number). The oval at the top of the graph indicates an overall mean change in MPR of -6% among the whole cohort (100%). Each split represents a dichotomization of the data with ‘yes’ on the lest and ‘no’ on the right. So, the first split was chosen by the algorithm as baseline MPR ≥43 (bl_mpr ≥ 43), those meeting the condition (‘yes’, on the left) had a mean decrease in MPR of -9.2 and comprised 87% of the cohort; those with baseline MPR <43 had a mean increase in MPR of 15 and represented the remaining 13% of the cohort. On the left side, those with baseline MPR ≥43 were then further subdivided by baseline SATMED effectiveness score ≥ 4. Those meeting this condition represented 76% of the cohort with a mean decrease in MPR of 11 vs. the 11% of the cohort with baseline SATMED effectiveness <4 with a mean 6-month MPR increase of 2.3.

## Data Availability

The study PI (Dr. Singh) and biostatistician (Dr. Richman) had access to the final trial dataset.

## Abbreviations

AA: African Americans
HBM: Health Belief Model
MEMS: Medication Event Monitoring System
MPR: Medication Possession Ratio
SU: Serum urate (serum uric acid)
SATMED-Q: Satisfaction with Medications – Questionnaire
ULT: Urate-lowering therapy
