# Supplementary figures and images for: SToRytelling to Improve Disease outcomes in Gout (STRIDE-GO): a multicenter, randomized controlled trial in African American veterans with gout
Source: BMC Med. 2021 Nov 9;19:265. doi: 10.1186/s12916-021-02135-w (PMC8576883; doi:10.1186/s12916-021-02135-w)

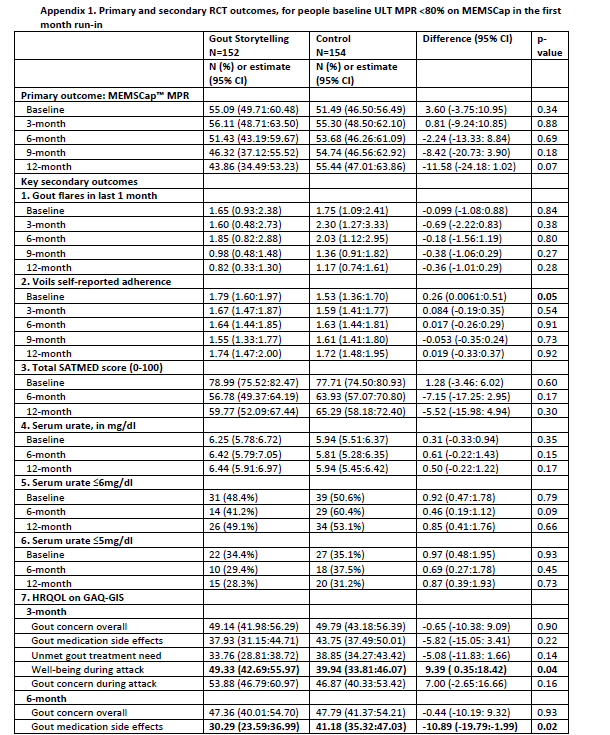


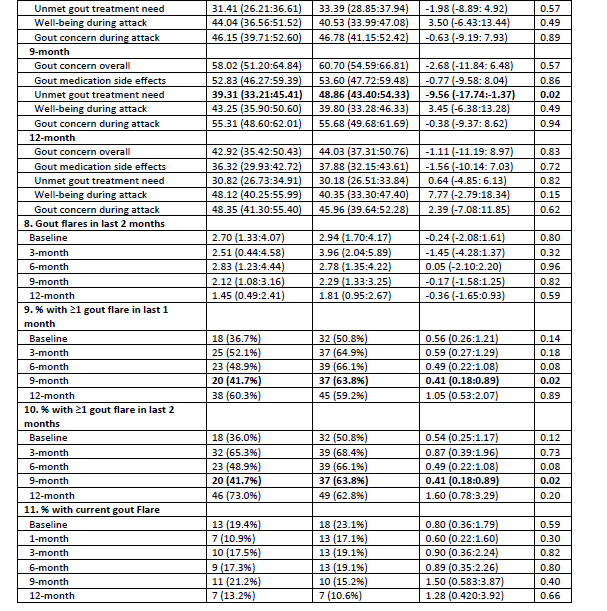

Supplement: Supplementary file 1 — Additional file 1.. Primary and secondary RCT outcomes, for people with baseline MEMSCap™ ULT MPR <80% during the study run-in period. The additional file provides primary and secondary outcomes data for people with low baseline ULT MPR, comparing gout storytelling to stress reduction ‘control’ intervention. [file 12916_2021_2135_MOESM1_ESM.docx]

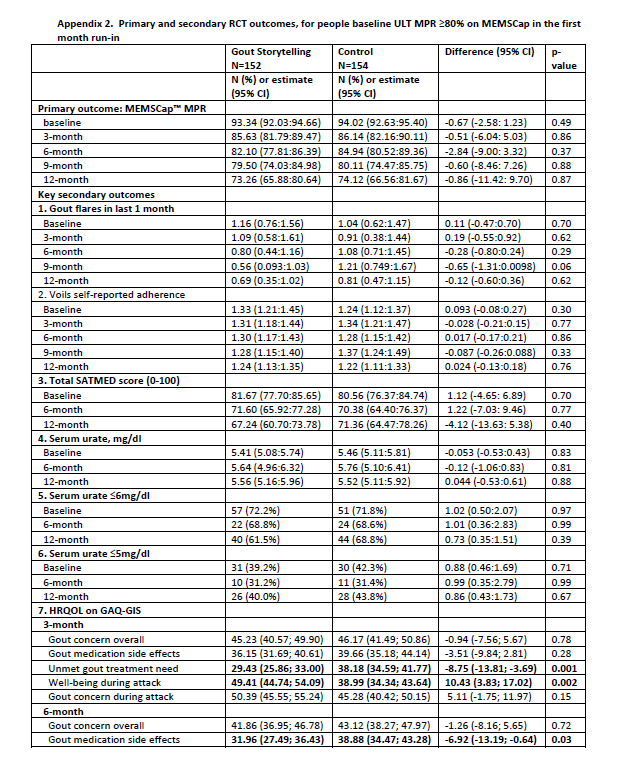


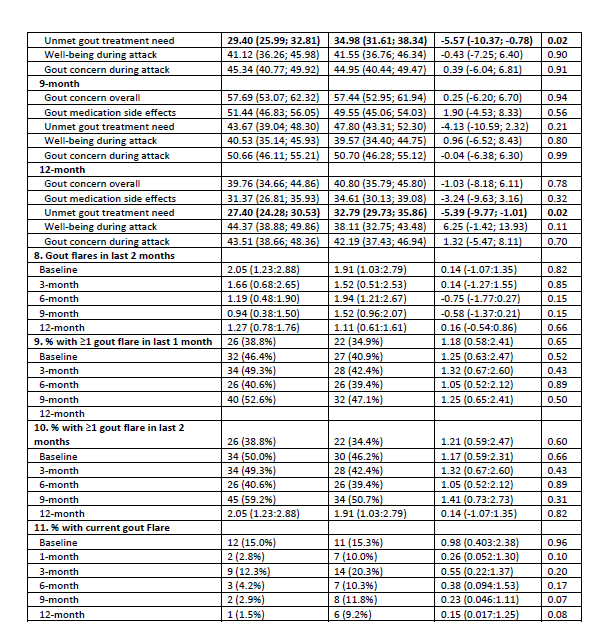

Supplement: Supplementary file 2 — Additional file 2.. Primary and secondary RCT outcomes, for people with baseline MEMSCap™ ULT MPR ≥80% during the study run-in period. The additional file provides primary and secondary outcomes data for people with high baseline ULT MPR, comparing gout storytelling to stress reduction ‘control’ intervention. [file 12916_2021_2135_MOESM2_ESM.docx]
