# Supplementary material for: SToRytelling to Improve Disease outcomes in Gout (STRIDE-GO): a multicenter, randomized controlled trial in African American veterans with gout
Source: BMC Med. 2021 Nov 9;19:265. doi: 10.1186/s12916-021-02135-w (PMC8576883; doi:10.1186/s12916-021-02135-w)
Supplement: Supplementary file 3 — Additional file 3:. Fig. S1. Regression tree for ULT MPR at 6 months. Regression tree results for ULT MPR at 6 months. The tree was allowed to consider all baseline variables that were used in any analyses. The goal was to identify whether there were any subgroups in which the intervention was efficacious. This would have been indicated by the appearance of ‘group’ at one of the split points in the tree. Our regression tree does not show ‘group’ at any split points in the tree. To interpret; each oval contains the mean MPR in that group (top number) and percent of the cohort (bottom number). The oval at the top of the graph indicates an overall mean MPR of 69% among the whole cohort (100%). Each split represents a dichotomization of the data with ‘yes’ on the left and ‘no’ on the right. So, the first split was chosen by the algorithm as baseline MPR < 78 (bl_mpr < 78), those meeting the condition (‘yes’, on the left) had a mean MPR of 51 and comprised 45% of the cohort; those with baseline MPR ≥78 had a mean MPR of 83 and represented the remaining 55% of the cohort. On the left side, those with baseline MPR <78 were then further subdivided by baseline MPR <55. Those meeting this condition represented 20% of the cohort with a mean MPR of 39 vs. the 24% of the cohort with baseline MPR ≥55 (but less than 78) with a mean 6-month MPR of 62. [file 12916_2021_2135_MOESM3_ESM.docx]

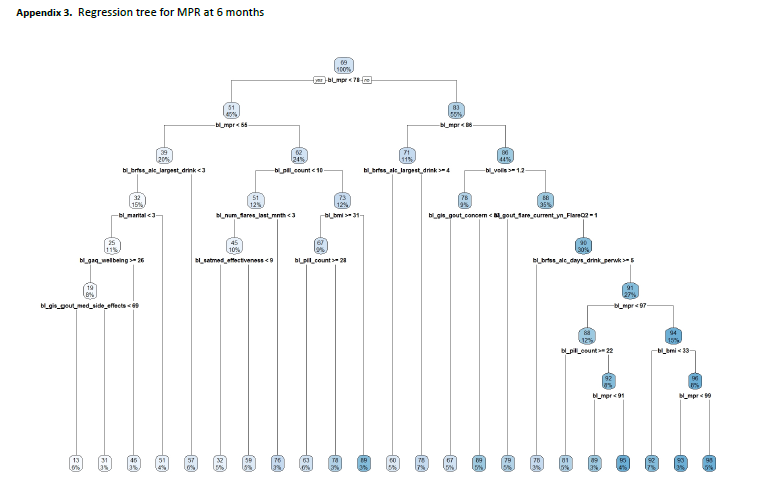


Legend for appendix 4: Regression tree results for ULT MPR at 6 months. The tree was allowed to consider all baseline variables that were used in any analyses. The goal was to identify whether there were any subgroups in which the intervention was efficacious. This would have been indicated by the appearance of ‘group’ at one of the split points in the tree. Our regression tree does not show ‘group’ at any split points in the tree. To interpret: each oval contains the mean MPR in that group (top number) and percent of the cohort (bottom number). The oval at the top of the graph indicates an overall mean MPR of 69% among the whole cohort (100%). Each split represents a dichotomization of the data with ‘yes’ on the left and ‘no’ on the right. So, the first split was chosen by the algorithm as baseline MPR < 78 (bl_mpr<78), those meeting the condition (‘yes’, on the left) had a mean MPR of 51 and comprised 45% of the cohort; those with baseline MPR ≥78 had a mean MPR of 83 and represented the remaining 55% of the cohort. On the left side, those with baseline MPR <78 were then further subdivided by baseline MPR <55. Those meeting this condition represented 20% of the cohort with a mean MPR of 39 vs. the 24% of the cohort with baseline MPR ≥55 (but less than 78) with a mean 6-month MPR of 62.
