# Supplementary material for: SToRytelling to Improve Disease outcomes in Gout (STRIDE-GO): a multicenter, randomized controlled trial in African American veterans with gout
Source: BMC Med. 2021 Nov 9;19:265. doi: 10.1186/s12916-021-02135-w (PMC8576883; doi:10.1186/s12916-021-02135-w)
Supplement: Supplementary file 4 — Additional file 4:. Fig. S2. Regression tree for change in ULT MPR at 6 months. Regression tree results for change in ULT MPR from baseline to 6 months, with negative change indicating a decrease in MPR. The tree was allowed to consider all baseline variables that were used in any analyses. The goal was to identify whether there were any subgroups in which the intervention was efficacious. This would have been indicated by the appearance of ‘group’ at one of the split points in the tree. Our regression tree does not show ‘group’ at any split points in the tree. To interpret; each oval contains the mean MPR in that group (top number) and percent of the cohort (bottom number). The oval at the top of the graph indicates an overall mean change in MPR of -6% among the whole cohort (100%). Each split represents a dichotomization of the data with ‘yes’ on the lest and ‘no’ on the right. So, the first split was chosen by the algorithm as baseline MPR ≥43 (bl_mpr ≥ 43), those meeting the condition (‘yes’, on the left) had a mean decrease in MPR of -9.2 and comprised 87% of the cohort; those with baseline MPR <43 had a mean increase in MPR of 15 and represented the remaining 13% of the cohort. On the left side, those with baseline MPR ≥43 were then further subdivided by baseline SATMED effectiveness score ≥ 4. Those meeting this condition represented 76% of the cohort with a mean decrease in MPR of 11 vs. the 11% of the cohort with baseline SATMED effectiveness <4 with a mean 6-month MPR increase of 2.3. [file 12916_2021_2135_MOESM4_ESM.docx]

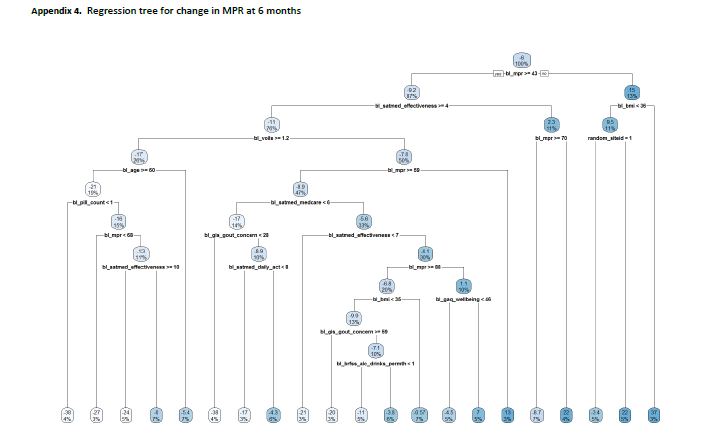


Legend for appendix 5: Regression tree results for change in ULT MPR from baseline to 6 months, with negative change indicating a decrease in MPR. The tree was allowed to consider all baseline variables that were used in any analyses. The goal was to identify whether there were any subgroups in which the intervention was efficacious. This would have been indicated by the appearance of ‘group’ at one of the split points in the tree. Our regression tree does not show ‘group’ at any split points in the tree. To interpret: each oval contains the mean MPR in that group (top number) and percent of the cohort (bottom number). The oval at the top of the graph indicates an overall mean change in MPR of -6% among the whole cohort (100%). Each split represents a dichotomization of the data with ‘yes’ on the lest and ‘no’ on the right. So the first split was chosen by the algorithm as baseline MPR ≥43 (bl_mpr≥43), those meeting the condition (‘yes’, on the left) had a mean decrease in MPR of -9.2 and comprised 87% of the cohort; those with baseline MPR <43 had a mean increase in MPR of 15 and represented the remaining 13% of the cohort. On the left side, those with baseline MPR ≥43 were then further subdivided by baseline SATMED effectiveness score ≥4. Those meeting this condition represented 76% of the cohort with a mean decrease in MPR of 11 vs. the 11% of the cohort with baseline SATMED effectiveness <4 with a mean 6-month MPR increase of 2.3.
